# Development and Validation of an Entrustable Professional Activity-Based Assessment Scale for Nurse Practitioners in Taiwan

**DOI:** 10.1097/jnr.0000000000000682

**Published:** 2025-06-23

**Authors:** Sophia Huey-Lan HU, Shiow-Luan TSAY, Sheng-Shiung HUANG, Heng-Hsin TUNG, Ying-Ru CHEN, Ling-Chun LU, Chi CHANG, Jia-Ying HU, Wei-Chieh HUNG

**Affiliations:** 1 School of Nursing, National Yang-Ming Chiao Tung University, Taipei, Taiwan; 2 College of Nursing and Health Sciences, Da-Yeh University, Chunghwa, Taiwan; 3 Department of Orthopedic Surgery, National Taiwan University Hospital, Taipei, Taiwan; 4 Second Degree Bachelor of Nursing, National Taiwan University, College of Medicine, Taipei, Taiwan; 5 Development, and Management Center, MacKay Memorial Hospital Tamsui Branch, Taipei, Taiwan; 6 Nurse Practitioner Department, Department Yuan's General Hospital, Kaohsiung City, Taiwan; 7 Department of Nursing, Far Eastern Memorial Hospital, New Taipei City, Taiwan

**Keywords:** nurse practitioners, entrustable professional activities, reliability, validity, fever

## Abstract

**Background:**

Competency-based education is essential for training nurse practitioners (NPs). Although entrustable professional activities (EPAs) have been widely used to assess competency in health professionals, a valid EPA-based assessment scale is required to assess the clinical competencies of NPs in acute care settings.

**Purpose:**

The aim of this study was to develop and examine the reliability and validity of an EPA-based assessment scale for NPs.

**Methods:**

A psychometric study with a cross-sectional survey was used in this study. The participants included NP instructors as evaluators and novice NPs currently in clinical practice as test takers. Convenience sampling was used to recruit participants from among members of the Taiwan Association of Nurse Practitioners. First, five EPA focus groups were used to develop five EPAs using a template and following the suggested steps. Second, a consensus validation was conducted using the Delphi study. Third, content validity was performed through a national study involving 218 novice NPs as test takers and 57 certified clinical NP educators serving as observers to test the EPAs. The Cronbach's alpha and intraclass correlation coefficient were calculated to examine EPA-based assessment scale reliability, and exploratory factor analysis, concurrent validity, and discriminant validity were applied to assess the validity of the EPAs. Finally, the EPA-based assessment scale of NP care for patients with fever was used in data analysis.

**Results:**

The final version of the EPA-based assessment scale included a 22-item observable checklist scale designed to evaluate the clinically independent performance (1–5) of nine key NP competencies. The Cronbach's alpha coefficient for the overall scale was .95. The results revealed that the EPA-based assessment scale addressed two key factors of direct patient-centered care and communication/time management. Factor loadings for each item ranged from .58 to .83, accounting for 70.83% of the total variance in the EPA-based assessment scale. Concurrent validity indicated a high correlation between the developed EPA-based assessment scale and the Ottawa Clinic Assessment Tool (*r* = .96, *p* < .001). The results of the discriminant validity analysis indicated a statistically significant difference between novice and expert NPs (*F* = 7.84, *p* < .001).

**Conclusions/Implications for Practice:**

The novel EPA-based assessment scale developed in this study demonstrated satisfactory reliability and validity, thereby supporting its application in evaluating the clinical competencies of NPs.

## Introduction

Nurse practitioner (NP) programs typically use competency-based education (CBE) to train NPs. The basic premise of CBE is that learners must demonstrate proficiency in target competencies prior to entering clinical practice. The American Association of Colleges of Nursing defines CBE as an educational approach in which individuals progress by demonstrating mastery of specific competencies or learning outcomes and that emphasizes applying knowledge, skills, and abilities as learners grow through the educational system. In CBE, instruction, assessment, feedback, self-reflection, and reporting are aligned with the competencies that individuals need to develop. Individuals advance at their own pace as they provide evidence of achieving required competencies (American Association of Colleges of Nursing, 2022). The numerous methods used to measure competencies for practice readiness often include clinical preceptor feedback, objective structured clinical examinations, MiniCEX, 360° evaluations, skill laboratories, and simulations (Hodges et al., 2019). However, NP programs continue to struggle to bridge the gap between theory and delivering care in clinical settings (Hodges et al., 2019).

In addition, research has documented NPs feel inadequately prepared for practice after completing basic NP training as novices (Chiang et al., 2022; Moore & Hawkins-Walsh, 2020; Yang et al., 2021). The novice NP is defined as a newly graduated NP with an NP license who has accumulated less than 1 year of clinical experience in practice. As a result, the majority of novice NPs entering healthcare settings often lack the skills necessary to care for patients and feel unprepared for independent practice. Furthermore, NPs in training do not acquire the skills necessary to care for patients at the same pace or time frame. In other words, NP training is based on the amount of time trainees have spent in the role rather than on their successful acquisition of key competencies. A potential approach to addressing these issues is to adopt a CBE model that incorporates entrustable professional activities (EPAs) to provide documented evidence of knowledge and skill acquisition (O'Dowd et al., 2020; ten Cate, 2013; ten Cate et al., 2016).

Clinical competence is essential for healthcare clinicians to be accountable and responsible for providing high-quality, safe patient care. The Institute of Medicine (2003) published an article calling for health professionals to demonstrate the following five core competencies in the delivery of patient care: (a) the ability to provide patient-centered care, (b) the ability to work in interdisciplinary teams, (c) the ability to use evidence-based practice, (d) the ability to apply quality improvement, and (e) the ability to use informatics. In response to this initiative, the American Association of Colleges of Nursing (2017) reported competencies in master's and doctoral education programs for advanced nursing practice. Furthermore, the Taiwan Association of Nurse Practitioners (TANP) has developed and documented competencies for NPs in clinical practice, encompassing the domains most relevant to providing patient-centered care. Collaboration, education, quality of care, and research, as well as leadership and professionalism, are also essential domains for NPs (Tsay et al., 2023).

EPAs are often used to assess clinical competence in healthcare education. Because EPAs integrate the cognitive, affective, and psychomotor domains of practice competency, they represent a holistic approach to clinical competence assessment (ten Cate & Favier, 2022). Entrustable professional activities are specific health professional practice tasks that capture the competencies in which NPs must become proficient before entering clinical practice independently. Each EPA contains several important competencies to the associated task, along with five developmental milestones that indicate the degree of supervision required by the trainee. These EPAs may be utilized to determine whether an NP can be trusted to perform a clinical task or if they still require supervision (ten Cate, 2013; ten Cate et al., 2016).

Despite the increasing use of EPA-based measures, the psychometric properties of EPAs have been inadequately tested. The resulting limited evidence for the validity of EPA-based entrustment scales may lead to bias in clinical competency assessments (Hodges et al., 2019; Meyer et al., 2022; Ryan et al., 2022).

There is considerable variation in how EPAs are developed. Although no one method has proven to be the most effective, common methods used to develop EPAs include the Delphi method, focus groups, and literature reviews (O'Dowd et al., 2019). In the literature, the process of developing EPAs often follows the guidelines provided by ten Cate et al. (2015), who suggested three broad steps to improving the EPA process, including initial development, expansion, and validation.

When describing an EPA, each component includes a title, a description of the EPA, relevant competency domains, required competencies, an assessment of progress, the process for making entrustment decisions, and an expiration date (ten Cate, 2013; ten Cate et al., 2016; Wagner et al., 2018). In this study, the recommendations of Hennus et al. (2023) and O'Dowd et al. (2020) were first combined to develop the proposed EPA in the following manner. First, a core team develops and uses an EPA template to construct the initial version of the EPAs. Next, the initial EPA content and structure are validated after modifying and expanding the initial EPAs. Subsequently, the levels of proficiency required with each initial EPA are confirmed, and experts are consulted to achieve a consensus on the process so far. Finally, if possible, the developed EPAs are benchmarked with a professional association.

The development of EPAs for NPs must target essential competencies that correspond to roles and functions. NPs provide direct care in collaboration with medical teams during the diagnosis and management of patients' health problems as well as act as advanced practice nurses on issues such as education, quality of care improvement, and professionalism (Tsay et al., 2023). Therefore, competency in caring for patients with common health issues is essential for NPs. Although several academic or clinical NP programs have developed EPA-based assessment tools for NP students and NPs, the validity and reliability of these tools have yet to be investigated (Chiang et al., 2022; Moore & Hawkins-Walsh, 2020; Surjadi et al., 2019; Yang et al., 2021). Given the absence of validated EPAs for assessing the clinical competency of NPs, this study was designed to develop an EPA-based assessment scale and to validate its use by NPs in clinical practice.

## Methods

### Study Design

Based on a cross-sectional survey, this psychometric study uses CBE as a framework for NP training. CBE focuses on standardized expected proficiency levels to ensure that all NP learners attain an appropriate level of proficiency at specific milestones throughout their educational process and training completion (ten Cate, 2013).

### Settings and Participants

A quantitative method was employed to test the developed EPAs for reliability and validity. In this study, NPs were recruited by certified clinical instructors to serve as evaluators. The participants were clinical NPs working in medical or surgical units. All participants in this study were recruited from the membership roster of TANP. Quantitative data for this study were collected from September 1 to November 30, 2023. The inclusion criteria for evaluators were NPs who (a) held national NP certification, (b) had worked in acute care settings for at least 5 years, and (c) were certified as NP clinical instructors. We invited eligible participants to take part in this study and provided detailed information about the study's purposes and procedures.

### Sample Size Estimation

The 218 NPs who completed the EPA-based assessment scale test was an effective sample size that met the minimum requirements for a two-factor model in exploratory factor analysis (EFA; recommended sample size: 110–220). The minimum sample size was estimated using the product of the factors and variables. The EPA-based assessment scale consists of 22 items, ideally including 5 to 10 observations for factor analysis (Mundfrom et al., 2005).

### Data Collection

Data for this study were collected from March 1 to December 31, 2023. Certified clinical NP instructors received training to serve as evaluators before utilizing the EPA to assess NPs' clinical competence in caring for patients with fevers. We recruited 57 certified clinical NP instructors as observers using newly developed EPAs from 18 major hospitals nationwide.

### Developing and Testing Entrustable Professional Activities

EPA development was based on the processes recommended by Hennus et al. (2023), O'Dowd et al. (2020), and ten Cate et al. (2015). First, an EPA template was selected by the research team, consisting of NP professors, educators, expert NPs, and physicians, which conducted a literature review and identified the key components of EPA templates. Second, five focus groups were formed, each consisting of six expert NPs and one physician. Using the NP core competencies of the TANP (Tsay et al., 2023) as a guide, the groups developed an initial list of five EPAs deemed important to NP practice after extensive discussion and agreement among the groups. Third, consensus validation was conducted using three rounds of the Delphi study, with the EPAs revised based on the results. Fourth, content validity was assessed with the involvement of nine NP experts and physicians, facilitating the subsequent modification of EPAs as needed. The five nested EPAs for NPs developed included assessment and caring protocols for patients with symptoms of fever, acute dyspnea, jaundice, headache, and low back pain. Finally, a national study that included 218 novice NPs as test takers and 57 qualified clinical NP instructors as evaluators was conducted nationwide using the newly developed EPAs to observe the clinical performance of NPs working at 18 hospitals. The EPA-based assessment scale was used to demonstrate reliability and testing validity.

### Testing the Reliability and Validity of the Entrustable Professional Activity-Based Assessment Scale

Internal consistency and interobserver reliability were tested to examine the reliability of the developed EPA-based assessment scale. Also, EFA, discriminatory validity, and concurrent validity were used to test the validity of the EPA-based assessment scale.

### Measurement

#### The entrustable professional activity-based assessment scale

**Figure 1:**
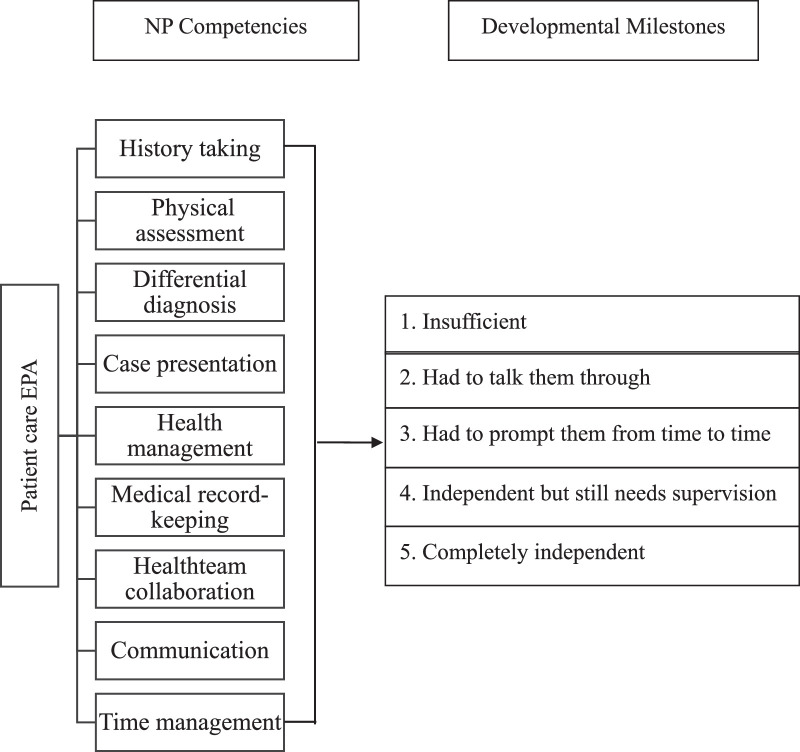
Entrustable Professional Activities Structure for NP Competencies and Milestones in Caring for Patients With Common Health Problems

**Figure 2:**
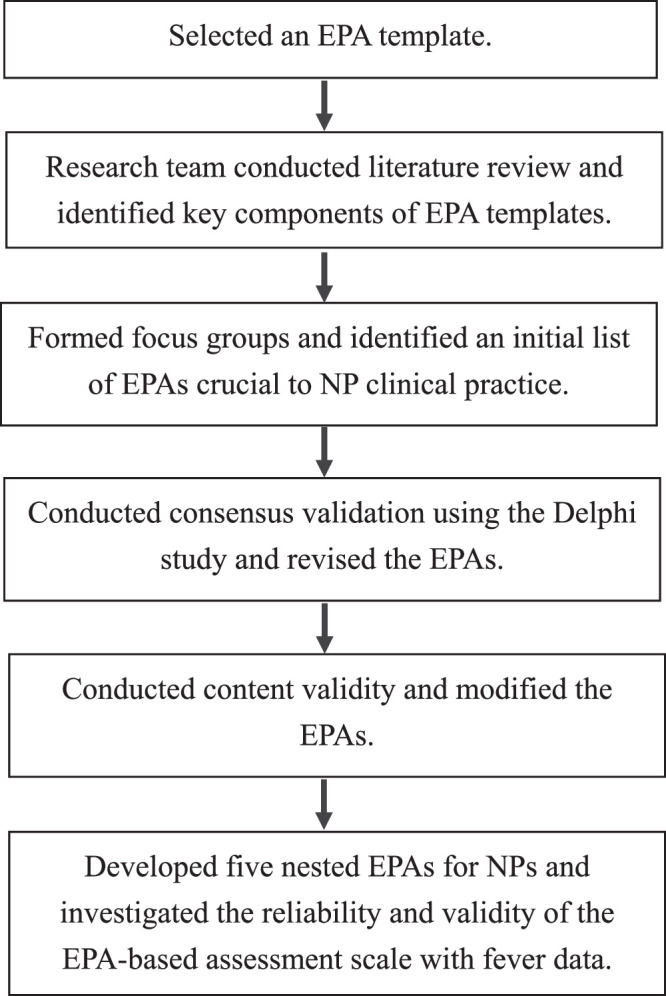
Entrustable Professional Activity-Based Assessment Scale Development Flowchart

A template was used to develop five nested EPAs all centering on the major NP competency of patient-centered care with illness symptoms. As the data collected from all five EPAs were shown to be similar, the EPA of fever was selected for the subsequent reliability and validity investigation. The EPA-based assessment scale is a 22-item observational behavior measure. Each item is scored between 1 and 5 for the level of performance, that is, insufficient (1), I had to talk them through (2), I had to prompt them from time to time (3), independent but still need supervision for safe practice (4), and completely independent (5). The scale covers NP competencies in history taking, physical assessment, differential diagnosis, health management, medical record keeping, health team collaboration, communication, and time management. The EPA-based assessment scale structure for NP competencies and milestones/levels of performance in patient care is shown in Figure 1. However, the EPA-based assessment scale may be used as a formative or summative evaluation method in the NP training process (Figure 2).

#### The Ottawa Clinic Assessment Tool

The Ottawa Clinic Assessment Tool (OCAT) was used in this study to test the concurrent validity of the newly developed EPA-based assessment scale. This tool was developed to assess clinical performance in residents. The OCAT contains nine items designed to measure the clinical competencies of residents, ranging in performance from 1 (*I had to do it*) to 5 (*I did not need to be there*). The nine mandatory items include clinical competencies in history-taking, physical examination, case presentation, differential diagnosis, management plan, patient/family communication, clinic documentation, collaboration, and time management. These competencies are similar to standard NP clinical practices. The OCAT has demonstrated interclass correlation ranging from .68 to .91, indicating good reliability. The validity test of OCAT using generalizability analysis revealed substantial variance in ratings attributable to the learner–EPA interaction (59.6% for Ottawa), which supports the validity of this tool (Ryan et al., 2022). Overall, the results support OCAT reliability and validity (Ryan et al., 2022).

### Ethical Considerations

Informed consent was obtained from all eligible participants before the study began. Potential participants were told they would be free to withdraw without condition at any time during the study period. This study was approved by the institutional review board of the hospital (No. EMRP-112-154).

### Data Analysis

Participant characteristics were analyzed using descriptive statistics, including counts (*n*), percentages (%), means (*M*), standard deviations (*SD*), and ranges. The normality of the data was assessed using skewness and kurtosis, with skewness values < 2 and kurtosis values < 4 (Tabachnick & Fidell, 2019).

Cronbach's alpha and the intraclass correlation coefficient (ICC) were used to evaluate the reliability of the EPA-based assessment scale, with Cronbach's alpha values above .70 indicating acceptable (Taber, 2018) and ICC values above .75 indicating good reliability (Koo & Li, 2016). EFA was conducted to assess construct validity. Both Bartlett's spherical test and the Keizer–Meyer–Olkin measure of sampling adequacy were performed to examine EFA suitability. The statistical significance of Bartlett's sphericity test and Keizer–Meyer–Olkin values greater than .5 confirmed data suitability for EFA (Hair et al., 2006). The potential factors were extracted using principal component analysis, and varimax with Kaiser normalization, the most common orthogonal rotation method, was used for rotation (Osborne, 2015). The number of factors was determined based on the count of eigenvalues exceeding 1, in conjunction with the variance accounted for by each factor, necessitating a factor loading of at least .40 (Costello & Osborne, 2005; Yong & Pearce, 2013). In addition, concurrent validity was assessed using the Pearson correlation coefficient, and the Kruskal–Wallis test was used to examine discriminant validity. The significance level (α) was set at < .05, and all statistical analyses were performed using SPSS Statistics 24.0 (IBM Inc., Armonk, NY, USA).

## Results

### Novice Nurse Practitioner Characteristics

The majority of the novice NP (test taker) participants were female (93.6%) and were of a mean age of 41.58 (*SD* = 7.25; range = 25–56) years. Most (84.9%) were educated to the university level, with 11.0% educated to the master's or PhD level. A majority (60.6%) practiced in an internal medicine department, and 39.4% practiced in a surgical department. Average experience as an NP was 7.60 (*SD* = 5.05) years.

### Evaluator Characteristics

The majority of evaluators were female (96.5%) and were of a mean age of 45.54 (*SD* = 5.27; range = 33–60) years. All evaluators held a university degree, and 31.6% held a master's or PhD degree. Most evaluators (66.70%) practiced in an internal medicine department, whereas 31.60% practiced in a surgical department. The average experience as an NP was 12.14 (*SD* = 3.89) years.

### Assessment and Management of Patients With Entrustable Professional Activity-Based Assessment Scale Results

The mean EPA score was 97.14 (*SD* = 13.93; range = 25–110), with higher scores indicating greater NP independence in clinical practice (Table 1). Scores for individual items on the EPA scale are also shown in Table 2. Although items 12, 13, 14, 17, and 21 exhibited slightly elevated skew and kurtosis values, the skew and kurtosis of all items met the conditions of normality (Table 2).

**Table 1:** Demographic Characteristics, Participants and Evaluators

Characteristic	*n*	%
**Novice NP participants (*n* = 218)**		
Age (mean and *SD*)	41.58	7.25
Gender		
Male	14	6.4
Female	204	93.6
Educational level		
College	9	4.1
University	185	84.9
Master/Doctor of Philosophy	24	11.0
Practicing department		
Internal medicine	132	60.6
Surgery	86	39.4
Years of experience (mean and *SD*)	7.60	5.05
Professional career ladder		
0	35	16.1
I	46	21.1
II	113	51.8
III	12	5.5
IV	2	0.9
V	10	4.6
**NP evaluators (*n* = 57)**		
Age (mean and *SD*)	45.54	5.27
Gender		
Male	2	3.5
Female	55	96.5
Educational level		
College	39	68.4
Master/Doctor of Philosophy	18	31.6
Practicing department		
Internal medicine	38	66.7
Surgery	18	31.6
Obstetrics and gynecology	1	1.8
Years of experience (mean and *SD*)	12.14	3.89

*Note*. NP = nurse practitioner.

**Table 2:** Descriptive Statistics for the 22 Entrustable Professional Activity Items (*N* = 218)

Item	Mean	*SD*	Range	Skew	Kurtosis
1. Rule out urgent conditions	4.00	1.31	1–5	−1.20	0.32
2. Identify condition for emergency management	4.18	1.01	1–5	−1.28	1.33
3. Seeks relevant data for the differential diagnoses	4.23	.87	1–5	−0.85	0.02
4. Conduct and interpret physical examination findings	4.33	.83	1–5	−1.03	0.41
5. Arrange examination, laboratory tests, and consultation	4.36	.80	1–5	−1.12	0.93
6. Making differential diagnoses	4.34	.80	1–5	−1.09	0.89
7. Interpret relevant examination and laboratory results	4.45	.78	1–5	−1.39	1.70
8. Clinical reasoning and differential diagnoses	4.32	.85	1–5	−1.20	1.26
9. Update the differential diagnoses	4.36	.83	1–5	−1.15	0.80
10. Prioritize the differential diagnoses	4.33	.85	1–5	−1.06	0.42
11. Prioritizes care decision	4.45	.75	1–5	−1.28	1.49
12. Communicate and update patient's condition	4.53	.73	1–5	−1.84	4.36
13. Providing patient management	4.44	.96	1–5	−2.12	4.52
14. Makes recommendations to patient safety	4.67	.64	1–5	−2.19	5.69
15. Provide record documentation	4.39	.78	1–5	−1.09	0.71
16. Complete an accurate and timely documentation	4.45	.76	1–5	−1.22	1.08
17. Effective communication	4.65	.61	1–5	−2.02	5.89
18. Demonstrate good communication skills	4.59	.68	1–5	−1.82	3.89
19. Collaborate with interprofessional team	4.64	.62	1–5	−1.64	2.06
20. Manage unexpected events timely	4.50	.74	1–5	−1.54	2.42
21. Organized care in a timely manner	4.59	.67	1–5	−1.91	4.81
22. Overall entrustable level	4.35	.76	1–5	−1.01	0.83
Total	97.14	13.93	25–110	−1.25	2.30

### Reliability of the Entrustable Professional Activity-Based Assessment Scale

#### Internal consistency

The Cronbach's α of the overall EPA was .95, with a coefficient of .94 for patient-centered care and .93 for communication and time management.

#### Interrater reliability

The ICC was calculated using a single-rater, absolute agreement, and two-way random effects model, with two evaluators simultaneously observing one novice NP (test taker). The ICC of the EPA-based assessment scale was .87 (95% CI [.62, .96], *F* = 14.35, *p* < .001).

### Validity of the Entrustable Professional Activity-Based Assessment Scale

#### Construct validity

*Exploratory factor analysis:* Correlation matrix analysis revealed all items to be significantly intercorrelated (ranging from .30 to .59). The results of the Bartlett's sphericity test met statistical significance (χ^2^ = 5288.91, *p* < .001), indicating adequate correlations among the items of the extracted factors. Furthermore, the Kaiser-Meyer-Olkin measure of sampling adequacy yielded a value of .95 (> .5), indicating the degree of partial correlation in the data to be sufficiently high to conduct an EFA. The principal factor analysis in the EFA identified two factors based on criteria such as scree plots and cumulative explained variance. The results of the EFA revealed two factors to be correlated with the factor items, with patient-centered care explaining 63.48% and communication/time management explaining 7.35% of the variance. Patient-centered care was defined in this study as NPs having adequate competencies in history taking, conducting physical examinations and differential diagnoses, and illness management. Communication/time management was defined in this study as NPs being able to communicate effectively during patient care activities. The 22 items explained a total of 70.83% of the variance (Table 3). The factor loading of each item was moderate to high (ranging from .58 to .83; Table 4).

**Table 3:** Entrustable Professional Activity Extraction Loading and Variance (*N* = 218)

Factor	Initial Eigenvalue	Rotation Sums of Squared Loading	
		Variance (%)	Cumulative Variance (%)
Patient-centered care	13.97	39.77	39.77
Communication and time management	1.62	31.06	70.83

**Table 4:** Factor Loadings for EPA Items (*N* = 218)

EPA Item	Factor Loading	
	1	2
Factor 1: Patient-centered care		
7. Interpret relevant examination and laboratory results	**.83**	.35
2. Identify condition for emergency management	**.82**	.16
5. Arrange examination, laboratory tests, and consultation	**.79**	.39
6. Making differential diagnoses	**.79**	.42
8. Clinical reasoning and differential diagnoses	**.78**	.43
9. Update the differential diagnoses	**.75**	.49
10. Prioritize the differential diagnoses	**.74**	.51
22. Overall entrustable level	**.73**	.52
4. Conduct and interpret physical examination findings	**.72**	.43
13. Providing patient management	**.71**	.25
1. Rule out urgent conditions	**.69**	.16
3. Seeks relevant data for the differential diagnoses	**.69**	.39
11. Prioritizes care decision	**.64**	.62
15. Provide record documentation	**.64**	.49
16. Complete an accurate and timely documentation	**.58**	.47
Factor 2: Communication/time management		
17. Effective communication	.24	**.83**
18. Demonstrate good communication skills	.29	**.83**
21. Organized care in a timely manner	.29	**.79**
19. Collaborate with interprofessional team	.34	**.77**
20. Manage unexpected events timely	.35	**.76**
12. Communicate and update patient's condition	.40	**.70**
14. Makes recommendations to patient safety	.36	**.68**

*Note*. EPA = entrustable professional activity. Factor loadings for specific factors are marked in bold.

#### Concurrent Validity

The correlation between the EPA and the OCAT was .96 (*p* < .001).

#### Discriminatory Validity

The average EPA-based assessment scale scores showed statistically significant differences across various professional career ladders (Kruskal–Wallis *H* = 26.74, *p* < .001).

## Discussion

The results indicate that the developed EPA-based assessment scale demonstrates good internal consistency, interrater reliability, concurrent validity, construct validity, and discriminant validity. Overall, this scale demonstrated satisfactory psychometric properties, supporting its use as an instrument to assess NP clinical competencies.

The developed EPA-based assessment scale and its two factors earned Cronbach's alpha values of .95, .94, and .93, respectively, indicating high internal consistency (Schmitt, 1996). The reliability of the EPA-based assessment scale was further enhanced and supported by interrater reliability. However, Cronbach's alpha values greater than .90, and interitem correlation values exceeding .70 may indicate the presence of interitem redundancy (Taber, 2018). Therefore, in future studies, researchers may work to refine the EPA-based assessment scale items.

This study used CBE as the framework for NP education and followed ten Cate et al.'s (2016) recommendations for developing EPAs. In addition, a rigorous design with multiple data sources was implemented, with multisite sampling supporting the generalizability of the results. Also, the developed EPA-based assessment scale was shown to discriminate between the performance of novice and experienced NPs. Thus, this scale may be used not only to assess NP competency in assessing and treating patients with fever but also as a template to assess their clinical performance in various patient conditions. Furthermore, the newly developed and validated EPA may be used as a benchmark for the NP Career Ladder Program, which has been previously recommended for the final phase of EPA-based assessment scale development (O'Dowd et al., 2020).

The 22 items of the EPA-based assessment scale included observable clinical behaviors associated with direct and indirect patient care. The EPA-based assessment scale aligns with the American Association of Nurse Practitioners' 2024 statement that the clinical role of NPs is to meet the needs of the patient and that their other roles serve to support and promote this clinical role. In this study, 15 of the 22 essential behaviors identified by the NP experts and physicians were associated with the nursing process, suggesting that clinical competency should be targeted in early NP development.

This study provides empirical evidence supporting the notion that the diagnostic process, although complex, may be translated into observable behaviors and assessed effectively. Cook and Décary (2020) found that diagnosing requires higher-order thinking, using higher cognitive processes, and integration beyond the simple memorization of facts and concepts. In addition, the seven non-nursing process behaviors identified, including communication, documentation, collaboration, and time management (efficiency), all relate to indirect patient care, requiring continuous improvement and supporting the ability of NPs to provide comprehensive services. NPs collect and analyze data to make decisions and report and discuss appropriate management plans and outcomes in a timely manner after completing training. Advancing medical technologies and the increasing complexity of health problems make increasing the education and training of NPs from the master's level to the level of doctor of nursing practice (DNP) extremely important and necessary to prepare them to be qualified clinicians (American Association of Nurse Practitioners, 2024).

To the best of the authors' knowledge, this was the first study to validate an EPA-based assessment scale for NP clinical competence in Taiwan. The results demonstrate the difference in competency levels between novice and experienced NPs. The Association of American Medical Colleges (2024) has issued a set of EPAs to guide the practice of medical residents in terms of both academic curriculum design and clinical performance evaluation (Bremer et al., 2022). As suggested by the Strong Model of Advance Practice Framework (Ackerman et al., 1996), the progression from novice to expert is a continuum leading from empowerment to collaboration to leadership. Novice NPs require supervision and a clear path to guide their professional practice and growth. Bremer et al. (2022) reported that EPAs help learners achieve better professional development by identifying their own learning opportunities and targeting their learning activities. There is a need to review NP requirements and to work with the TANP and expert colleagues to identify the most relevant EPAs for desired performance and nursing outcomes.

A milestone-based approach may be an appropriate next step in assessing NP performance to ensure core competencies have been achieved (Albright et al., 2020). Although evaluators use EPAs to rate levels of supervision based on levels of novice NP competency, milestones, that is, indicators of novice NP progress toward competency, may also be used to guide their “developmental levels” (Hart et al., 2019). For example, a first-year medical NP is expected to exhibit a minimum of level 3 competency in the designated core EPAs for medical NPs. By the end of their second year, they should achieve at least level 5 (independent) proficiency, and this expectation applies reciprocally. This may be expected to ensure novice NPs are on the right developmental track and progressing well.

Competency-based education, EPAs, and milestones have been increasingly used in healthcare-related education and on-the-job training over the past two decades and have been specifically recommended for NPs (Chan et al., 2020). Although EPAs and milestones have yet to be formally developed for NPs in Taiwan, the EPA-based assessment scale developed in this study offers a tool for both educators and supervisors to evaluate and design effective related training programs. Nursing faculty and leaders should be educated on designing EPAs that strongly reflect the core competencies of NP and on the application of EPAs to assess core competencies.

### Strengths and Limitations

A reliable and valid EPA-based assessment scale was developed in this study to measure clinical competency in novice NPs. This study's strengths include using both qualitative and quantitative processes in scale development, using observational items, and the thorough testing of scale psychometric properties. The developed EPA-based assessment scale identified two factors that explained over 70% of the total variance. Two types of reliability and three types of validity were obtained for the EPA-based assessment scale. However, several limitations of this study should be considered. First, the newly developed EPAs and evaluation scales for NPs in Taiwan have not yet been formally implemented in practice. Nonetheless, the eight EPAs addressed in the developed model were formulated by 45 expert NPs, 5 nursing scholars, and 2 physicians over a more than 2-year period in clinical settings. Furthermore, these EPAs were tested in this study for reliability and validity in 18 hospitals on novice NPs, with the results supporting the reliability and validity of the developed scale. However, how other NPs and hospitals will receive the scale is unknown. The collaboration of NP experts, physicians, and the TANP in the scale development process is expected to help promote scale implementation to a wider audience.

Second, the EPAs in this study were developed specifically for Taiwanese NPs in clinical practice. Developing country-specific EPAs may limit their generalizability to international health systems. However, most current EPA-based assessment scale development efforts are focused on a single country/culture. Moreover, this study is relatively unique due to the focus of the developed scale on novice NPs.

A final limitation is that the EPAs in this study were developed specifically for the acute care context in Taiwan, potentially limiting their applicability/generalizability to primary care settings.

### Conclusions/Implications for Practice

In this study, five EPAs for NPs in Taiwan were developed in collaboration with the TANP using a process of iterative consensus building. These EPAs were subsequently validated and demonstrated to have adequate reliability and validity. These EPAs and clinically based assessment tools must be tested in clinical contexts nationwide to ensure they fit their intended purposes and, once introduced to novice nurses and students, help NPs acquire the core competencies necessary to ensure their provision of safe and quality care to patients. It is essential for faculty and nursing leaders to acquire the skills necessary to design EPAs and integrate them into educational programs aimed at enhancing NP competencies, thereby fostering the delivery of high-quality patient care.
